# Trends in the global burden of anemia associated with liver cirrhosis: an assessment from 1990 to 2021 and projections to 2045

**DOI:** 10.3389/fpubh.2026.1845819

**Published:** 2026-07-15

**Authors:** Huili Ren, Jun Guan, Shaohui Zhang

**Affiliations:** 1 Department of Pharmacy, Traditional Chinese and Western Medicine Hospital of Wuhan, Tongji Medical College, Huazhong University of Science and Technology, Wuhan, China; 2 Department of Hematology, Traditional Chinese and Western Medicine Hospital of Wuhan, Tongji Medical College, Huazhong University of Science and Technology, Wuhan, China

**Keywords:** anemia, global burden of disease study, liver cirrhosis, prevalence, years lived with disability

## Abstract

**Background:**

As a major global health challenge, liver cirrhosis is accompanied by anemia that correlates closely with increased mortality. This study comprehensively assesses the burden of anemia associated with liver cirrhosis across global, regional, and demographic dimensions.

**Methods:**

Using Global Burden of Disease 2021 data, prevalence and years lived with disability (YLDs) of anemia associated with liver cirrhosis were analyzed globally, across five sociodemographic index (SDI) regions, and 21 geographical areas. Analyses were stratified by age, sex, and SDI. Joinpoint regression estimated average annual percentage changes (AAPC). Decomposition analysis identified drivers of burden trends, and the Nordpred model projected future trends to 2045.

**Results:**

In 2021, there were 644,273 (95% UI: 558,176–740,430) global cases of anemia associated with liver cirrhosis, marking a 26.02% rise from 1990. Meanwhile, YLDs reached 21,046 (13,156–30,926), corresponding to an 8.04% increase. However, age-standardized prevalence and YLDs rates declined significantly (AAPC: −0.82% and −1.08%). The greatest increases in absolute burden occurred in low SDI regions. Prevalence peaked in the 90–95 age group for both sexes. Decomposition revealed population growth (171.48%) as the primary driver of increased prevalence, while epidemiological changes (−112.36%) reduced rates. Projections to 2045 indicate continued declines in age-standardized rates but persistently high absolute burden due to population growth.

**Conclusion:**

Despite overall declines in age-standardized burden, the absolute prevalence and YLDs of anemia associated with liver cirrhosis are expected to rise. Continued focus on high-risk groups and region-specific prevention strategies is needed.

## 1 Introduction

Globally, more than 45 million patients are diagnosed with liver cirrhosis each year, resulting in 2 million deaths (1). Cirrhosis represents the advanced pathological stage of various chronic liver diseases. It not only results in progressive hepatic dysfunction but also induces a series of complex systemic pathophysiological alterations, involving metabolic, immune, circulatory, and hematological systems (2). Anemia is a common and serious systemic complication in patients with cirrhosis. It compromises tissue oxygen delivery, impairs daily functioning, increases the hepatic burden, elevates the risk of other complications, and contributes to poorer prognosis.

Evidence suggested that anemia appeared at any stage of cirrhosis, with a higher prevalence in patients with decompensated period (3). A prospective cohort that included 213 patients with compensated cirrhosis followed for 9 years reported a baseline prevalence of anemia of 21.1% (4). The clinical significance of anemia in the progression of liver cirrhosis has attracted growing attention. Studies have demonstrated that anemia was closely associated with adverse outcomes in patients with liver cirrhosis (3, 5–7). For patients with liver cirrhosis, every 10 g/L decrease in hemoglobin level increased the 90-day mortality risk by 6.8% and the 1-year mortality risk by 5.7% (6). Severe anemia was considered as an independent risk factor for poor prognosis in patients with liver cirrhosis complicated by acute decompensation and acute liver injury (6). The incidence of acute-on-chronic liver failure was significantly higher in patients with anemia than in those without anemia. This was because low hemoglobin concentration was independently associated with cerebral hypoxia and led to liver failure in patients with decompensated liver cirrhosis (8). Furthermore, different types of anemia mechanisms were also associated with the poor prognosis of liver cirrhosis. Megaloblastic anemia was related to the severity of liver damage. It predicted short-term mortality in patients with hepatitis B-related decompensated liver cirrhosis (9). Iron deficiency anemia common in liver cirrhosis was also significantly associated with an increased risk of death (10).

Despite the established negative impact of specific anemia types on cirrhosis prognosis, research on the population-level epidemiological profile and associated disease burden of anemia in cirrhosis remains inadequate. A comprehensive investigation into the epidemiological changes, components of disease burden, and factors driving regional variations in cirrhosis related anemia is essential to enhance the efficiency of health resource allocation. Therefore, this study systematically evaluates the global and regional trends and disease burden of anemia associated cirrhosis to provide an evidence-based foundation for developing precise and equitable prevention and control strategies.

## 2 Methods

### 2.1 Data source

The data used in this study were download from the GBD 2021, which provided a comprehensive estimation of the disease burden for 371 diseases and injuries, disaggregated by sex, age, region, ang country (11). The GBD Study utilizes data from the Global Health Data Exchange (GHDx),^1^ which includes census records, scientific literature, environmental monitoring, and disease registries. Each disease or injury is linked to a set of relevant sequelae (i.e., specific health outcomes or consequences) based on the GBD 2021. Years lived with disability (YLDs) for each disease and injury were estimated by multiplying the prevalence of each sequela (stratified by cause, age, sex, location, and year) by its disability weight. The study focused on the key metrics including prevalence, YLDs, age-standardized prevalence rates (ASPR) and age-standardized YLDs rates (ASYR) associated with cirrhosis attributable anemia. All rates, including age-standardized rates (ASRs), were calculated as the number of cases per 100,000 individuals and are presented accompanied by their 95% uncertainty intervals (UIs). Countries were classified into five quintiles based on the sociodemographic index (SDI), a composite measure of socioeconomic development, to investigate gradients in anemia burden by socioeconomic status. The GBD study rigorously adheres to the guidelines for accurate and transparent health estimates reporting (GATHER) statement (12).

### 2.2 Disease definitions

Anemia is defined by decreased blood concentration of hemoglobin, including < 110 g/L for children aged 6–59 months and pregnant women, < 115 g/L for children aged 5–11 years of age, < 120 g/L for children aged 12–14 years and non-pregnant women (15 years of age and above), and < 130 g/L for men (15 years of age and above). According to the International Classification of Diseases (ICD), anemia are coded as K70–K76, and cirrhosis are coded as D50–D64. In the GBD study, ‘anemia associated with liver cirrhosis’ is defined as the co-occurrence of cirrhosis (ICD-10 codes K70–K76) and anemia (D50–D64) where cirrhosis is identified as the underlying cause of anemia. The GBD team estimates the prevalence and mortality of this condition using a Bayesian meta-regression tool (DisMod-MR) applied to inpatient, outpatient, and vital registration data. A detailed definition is available in the GBD 2021 Anemia Collaborators’ protocol (13).

We extracted data from the GBD 2021 Anemia Prevalence by Cause dataset. The exact filter is: GBD_Estimatie = “Impairment”; Impairment _name = “Anemia”; Cause_name = “Cirrhosis.”

### 2.3 Joinpoint regression analysis

Temporal trends in prevalence and YLDs were analyzed using joinpoint regression analysis. The analysis identifies inflection points at which a statistically significant change in the linear slope of the trend occurs. To balance model fit and complexity, the maximum number of joinpoints was set to 5, and each segment was required to include at least 2 observed time points. The optimal number and spatial location of joinpoints were determined through the grid search method with the Bayesian Information Criterion (BIC) or the weighted BIC as the selection criterion. Model optimization was subsequently conducted using a Monte Carlo permutation test, thereby ensuring the statistical robustness of the identified temporal trends. The direction and magnitude of trends in each segment were assessed using the annual percent change (APC). To synthesize an overall trend measure, the average annual percent change (AAPC), a weighted mean of the segment APCs, was derived. Both metrics are reported as percentages with 95% confidence intervals (CIs). An upward trend is indicated by a value >0%, a downward trend by <0%, and stability is inferred when the 95% CI includes zero.

### 2.4 Decomposition analysis

A decomposition analysis based on the Das Gupta method was performed to quantify contributions of population growth, aging, and epidemiological changes to the global burden change from 1990 to 2021 (14). We used 20 age groups (5-year intervals, <5 to ≥95 years) and applied the standard Das Gupta weighting scheme (1/3 and 1/6 weights). This decomposition approach has been documented in prior studies (15). Specifically, we utilized a formula that considers the proportion of the population in different age categories, the total population, and the prevalence rate for specific age groups to calculate the prevalence for each year. By holding other variables constant, this methodology enabled us to quantify the distinct contributions of population growth, aging, and epidemiological shifts to the epidemiology of anemia caused by cirrhosis.

### 2.5 Prediction analysis

To project the burden of anemia caused by cirrhosis in the global from 2022 to 2045, the Nordpred package was used to estimate future prevalence, YLDs and their ASRs. The Nordpred method is an age-period-cohort predictive tool based on a generalized linear model framework. The model posits that disease rates are a function of three key factors: an individual’s age, the time period (period effects), and their birth cohort. It enables the precise assessment of independent age, period, and cohort effects on disease trends (16). Additionally, its shrinkage mechanism avoids unrealistic extrapolation beyond the observed period, which is especially valuable when projecting to 2045.

### 2.6 Statistical analysis

Throughout the analysis, data were processed and visualized using R software (version 4.4.1). Statistical significance was determined at a *p* < 0.05. Ethical approval was not required, as the research involved only secondary analysis of the public GBD database.

## 3 Results

### 3.1 Global burden of anemia associated with liver cirrhosis in 1990 and 2021

Globally, the prevalence and YLDs for anemia associated with liver cirrhosis showed significant increases from 1990 to 2021. The prevalence cases increased from 644,273 (95% UI: 558,176 to 740,430) in 1990 to 811,937 (95% UI: 714,089 to 916,698) in 2021 (Table 1), with an increased percentage of 26.02%. The YLDs increased from 21,046 (95% UI: 13,156 to 30,926) in 1990 to 22,738 (95% UI: 14,318 to 33,508) in 2021 (Table 2), with a percentage of 8.04%. In contrast, the ASPR and ASYR exhibited downward trends between 1990 and 2021. The ASPR was 12.98 per 100,000 (95% UI: 11.36 to 14.62) in 1990 and 10.06 per 100,000 (95% UI: 8.80 to 11.38) in 2021, with a global decline of −0.82% (95% CI: −0.84 to −0.80) over the 30-year period (Table 1). The ASYR decreased from 0.41 per 100,000 (95% UI: 0.26 to 0.60) in 1990 to 0.29 per 100,000 (95% UI: 0.18 to 0.43) in 2021, with an average decline of −1.08% (95% CI: −1.12 to −1.05; Table 2).

**Table 1:** Absolute prevalence and ASPR of anemia caused by cirrhosis in 1990 and 2021, and AAPC from 1990 to 2021.

Location	Prevalence (95% UI)		Age-standardized prevalence rates per 100,000 (95% UI)		1990–2021 AAPC (%, 95 CI)
	1990	2021	1990	2021	
Global	644,273(558176–740,430)	811,937(714089–916,698)	12.98(11.36–14.62)	10.06(8.8–11.38)	−0.82(−0.84 to −0.8)*
Sex					
Female	347,983(299528–399,671)	415,550(362155–470,525)	13.48(11.77–15.34)	10.32(8.93–11.81)	-0.82(−0.87 to −0.77) *
Male	296,290(257705–338,603)	396,388(347237–447,612)	12.6(11–14.23)	9.9(8.69–11.19)	−0.77(−0.83 to −0.71) *
SDI					
Low SDI	75,308(63669–88,949)	129,471(107088–154,186)	16.01(14.02–18.23)	12.68(10.84–14.59)	−0.76(−0.89 to −0.63) *
Low-middle SDI	193,418(165633–223,588)	274,794(236515–313,943)	17.62(15.38–19.96)	15.25(13.28–17.25)	−0.53(−0.59 to −0.48) *
Middle SDI	198,066(169074–230,359)	214,897(188535–243,784)	13.26(11.53–15.07)	8.47(7.42–9.57)	−1.43(−1.45 to −1.41) *
High-middle SDI	104,667(91219–119,951)	113,853(99495–128,923)	10.11(8.88–11.62)	7(6.19–7.94)	−1.23(−1.27 to −1.18) *
High SDI	72,235(62542–82,459)	78,270(67119–89,618)	7.1(6.15–8.08)	4.64(3.98–5.24)	−1.35(−1.39 to −1.3) *
Regions					
Australasia	347(280–430)	745(620–881)	1.57(1.27–1.94)	1.6(1.33–1.88)	0.06(−0.17 to 0.27)
North Africa and Middle East	41,664(34832–49,266)	63,633(54654–73,644)	14.43(12.65–16.33)	11.99(10.26–13.69)	−0.71(−1.09 to −0.42) *
Oceania	414(339–506)	943(799–1,127)	7.65(6.56–8.82)	7.64(6.56–8.92)	0.04(−0.03 to 0.12)
Southeast Asia	84,301(70968–97,921)	69,419(60459–79,339)	20.55(17.69–23.54)	10.18(8.92–11.55)	−2.19(−2.35 to −1.98) *
Eastern Europe	21,707(17937–26,701)	43,748(35862–53,356)	8.31(6.93–10.2)	15.43(12.72–18.93)	2.05(2.01 to 2.1) *
East Asia	111,118(93008–129,667)	73,595(61659–86,968)	10.47(8.76–12.31)	3.64(3.12–4.22)	−3.37(−3.45 to −3.33) *
High-income North America	19,278(16058–22,564)	29,559(23923–35,484)	5.88(4.87–6.91)	5.31(4.34–6.32)	−0.32(−0.37 to −0.28) *
High-income Asia Pacific	23,012(18452–28,172)	19,548(15842–24,310)	11.58(9.42–14.09)	5.32(4.38–6.33)	−2.5(−2.57 to −2.43) *
Caribbean	3,049(2527–3,590)	5,291(4694–6,037)	9.61(8.05–11.16)	10.82(9.48–12.47)	0.26(0.11 to 0.38) *
Andean Latin America	4,071(3243–5,043)	4,190(3538–4,940)	11.89(9.93–13.98)	6.94(5.87–8.13)	−1.34(−1.68 to −1) *
Southern Latin America	3,658(3000–4,476)	4,438(3696–5,368)	7.7(6.31–9.36)	5.46(4.53–6.64)	−1.37(−1.57 to −1.17) *
Tropical Latin America	16,912(13190–21,403)	13,499(10506–17,501)	14.65(11.43–18.44)	5.27(4.11–6.79)	−3.31(−3.43 to −3.21) *
Central Latin America	11,344(9724–13,076)	14,137(12290–16,112)	8.98(7.75–10.14)	5.61(4.91–6.39)	−1.53(−1.58 to −1.47) *
Southern Sub-Saharan Africa	3,223(2522–4,073)	3,182(2521–3,974)	7.26(5.92–8.89)	4.41(3.59–5.42)	−1.53(−1.69 to −1.35) *
South Asia	186,512(155822–222,495)	297,025(250483–347,641)	16.64(14.18–19.33)	16.52(14.01–19.17)	−0.05(−0.08 to −0.02) *
Eastern Sub-Saharan Africa	23,083(19587–27,301)	36,630(30980–43,082)	15.66(13.66–17.84)	11.48(10.03–13.05)	−1(−1.32 to −0.67) *
Western Sub-Saharan Africa	30,283(24889–36,228)	70,711(59721–83,264)	16.72(14.25–19.36)	15.96(13.95–18.23)	−0.17(−0.21 to −0.13) *
Western Europe	25,516(22450–28,666)	19,300(16849–21,864)	5.24(4.59–5.9)	2.68(2.33–3.07)	−2.21(−2.31 to −2.14) *
Central Sub-Saharan Africa	6,883(5480–8,606)	8,283(5955–10,917)	15.04(12.56–17.94)	6.69(5.02–8.55)	−2.54(−2.7 to −2.42) *
Central Europe	16,583(14435–18,689)	15,455(13359–17,556)	12.16(10.54–13.86)	9.66(8.34–11.09)	−0.77(−0.79 to −0.74) *
Central Asia	11,312(9739–13,014)	18,604(16551–21,003)	16.96(14.98–19.05)	20.79(18.59–23.3)	0.69(0.64 to 0.74) *

ASPR, age-standardized prevalence rate; AAPC, annual percentage changes, *p < 0.05.

**Table 2:** Absolute YLDs and ASYR of anemia caused by cirrhosis in 1990 and 2021, and AAPC from 1990 to 2021.

Location	YLDs (95% UI)		Age-standardized YLDs rates per 100,000 (95% UI)		1990–2021 AAPC (%, 95 CI)
	1990	2021	1990	2021	
Global	21,046(13156–30,926)	22,738(14318–33,508)	0.41(0.26–0.6)	0.29(0.18–0.43)	−1.08(−1.12 to −1.05) *
Sex					
Female	13,681(8602–20,145)	15,077(9609–21,995)	0.52(0.33–0.76)	0.38(0.24–0.57)	−0.95(−1.01 to −0.88) *
Male	7,365(4635–10,879)	7,661(4793–11,625)	0.3(0.19–0.45)	0.2(0.12–0.3)	−1.34(−1.41 to −1.26) *
SDI					
Low SDI	3,609(2270–5,310)	4,807(3036–7,177)	0.72(0.46–1.04)	0.45(0.28–0.66)	−1.51(−1.62 to −1.4) *
Low-middle SDI	8,378(5263–12,118)	9,428(6063–13,939)	0.74(0.48–1.05)	0.52(0.34–0.77)	−1.1(−1.14 to −1.07) *
Middle SDI	5,800(3628–8,603)	5,329(3290–7,873)	0.38(0.24–0.56)	0.22(0.14–0.33)	−1.82(−1.88 to −1.78) *
High-middle SDI	2,328(1490–3,484)	2,144(1338–3,232)	0.23(0.15–0.34)	0.14(0.09–0.2)	−1.69(−1.76 to −1.63) *
High SDI	918(543–1,495)	1,017(600–1,654)	0.09(0.05–0.15)	0.06(0.04–0.1)	−1.32(−1.37 to −1.26) *
Regions					
Australasia	5(3–8)	10(6–16)	0.02(0.01–0.04)	0.02(0.01–0.03)	−0.18(−0.38 to 0.02)
North Africa and Middle East	1,369(872–2025)	1701(1089–2,514)	0.45(0.29–0.66)	0.32(0.21–0.47)	−0.71(−1.09 to −0.42) *
Oceania	11(6–16)	21(13–34)	0.19(0.12–0.28)	0.17(0.11–0.27)	−0.34(−0.44 to −0.23) *
Southeast Asia	2084(1281–3,168)	1,455(902–2,159)	0.5(0.31–0.75)	0.22(0.14–0.33)	−2.43(−2.58 to −2.21) *
Eastern Europe	469(288–702)	802(499–1,207)	0.18(0.11–0.27)	0.29(0.18–0.43)	1.52(1.47 to 1.57) *
East Asia	2,697(1696–4,037)	1,333(811–2048)	0.27(0.17–0.41)	0.07(0.04–0.11)	−4.34(−4.41 to −4.29) *
High-income North America	232(139–383)	372(217–601)	0.07(0.04–0.12)	0.07(0.04–0.11)	−0.09(−0.14 to −0.04) *
High-income Asia Pacific	217(119–368)	224(128–353)	0.11(0.06–0.19)	0.05(0.03–0.09)	−2.42(−2.47 to −2.37) *
Caribbean	82(50–127)	131(82–197)	0.24(0.15–0.37)	0.28(0.17–0.43)	0.3(0.13 to 0.43) *
Andean Latin America	108(62–173)	82(49–124)	0.3(0.18–0.46)	0.14(0.08–0.21)	−1.99(−2.35 to −1.62) *
Southern Latin America	45(27–71)	51(30–84)	0.1(0.06–0.15)	0.06(0.04–0.1)	−1.71(−1.93 to −1.49) *
Tropical Latin America	361(221–569)	246(147–391)	0.3(0.19–0.47)	0.1(0.06–0.16)	−3.65(−3.85 to −3.5) *
Central Latin America	267(165–414)	240(149–381)	0.19(0.12–0.29)	0.1(0.06–0.15)	−2.2(−2.26 to −2.14) *
Southern Sub-Saharan Africa	108(65–167)	95(57–147)	0.22(0.14–0.34)	0.13(0.08–0.19)	−1.75(−1.91 to −1.55) *
South Asia	9,291(5821–13,573)	10,744(6745–15,932)	0.83(0.54–1.19)	0.61(0.39–0.9)	−0.99(−1.03 to −0.95) *
Eastern Sub-Saharan Africa	925(597–1,345)	1,089(668–1,617)	0.56(0.36–0.81)	0.32(0.2–0.46)	−1.78(−2.15 to −1.36) *
Western Sub-Saharan Africa	1,414(898–2,110)	2,866(1835–4,250)	0.71(0.45–1.04)	0.59(0.38–0.85)	−0.64(−0.72 to −0.57) *
Western Europe	300(182–487)	244(146–392)	0.06(0.04–0.1)	0.03(0.02–0.05)	−2.06(−2.2 to −1.95) *
Central Sub-Saharan Africa	288(177–431)	249(146–389)	0.59(0.37–0.86)	0.19(0.11–0.3)	−3.71(−3.98 to −3.52) *
Central Europe	336(211–502)	240(146–371)	0.25(0.16–0.37)	0.15(0.09–0.23)	−1.68(−1.7 to −1.67) *
Central Asia	437(279–661)	543(344–806)	0.62(0.4–0.93)	0.59(0.38–0.88)	−0.12(−0.18 to −0.07) *

YLDs, years lived with disability; ASYR, age-standardized YLDs rate; AAPC, annual percentage changes, *p < 0.05.

### 3.2 SDI and 21 regional trend analysis

From 1990 to 2021, the prevalence of anemia had risen in all five SDI regions, exhibiting a greater percentage increase in regions with lower SDI levels (Table 1). Among them, the low SDI region exhibited the largest increase from 75,308 (95% UI: 63,669-88,949) in 1990 to 129,471 (95% UI: 107,088 to 154,186), with an increased percentage of 71.92%. While, the high SDI region exhibited the smallest change, increasing only 8.35% from 72,235 (95% UI: 62,542 to 82,459) in 1990 to 78,270 cases (95% UI: 67,119 to 89,618) in 2021. Conversely, the ASPR across regions with different SDI levels showed a negative trend. Notably, the decline was most pronounced in the middle SDI region, with an AAPC of −1.43% (−1.45 to −1.41). Furthermore, the AAPC in the high-middle and high SDI regions was −1.23% (−1.27 to −1.18) and −1.35% (−1.39 to −1.30; Table 1), respectively. These findings indicated that the rate of decline in disease burden was heterogeneous across regions with varying development levels, exhibiting a nonlinear gradient pattern, with the most rapid downward trend observed in the middle SDI region.

The change in YLDs was not uniform across the SDI. The middle and high middle SDI regions experienced reductions in YLDs (Table 2). In contrast, increases were observed in the low, low middle, and high SDI regions. Among these, the increase was most pronounced in the low SDI region, where the value rose from 3,609 (95% UI: 2,270 to 5,310) in 1990 to 4,807 (95% UI: 3,036 to 7,177) in 2021. A significant decline in ASYR was noted for all SDI regions. Notably, the middle SDI region showed the most pronounced decline, with an AAPC of −1.82% (95% CI: −1.88 to −1.78), representing the largest reduction among all SDI regions (Table 2).

Compared to 1990, the majority of GBD regions exhibited consistent increases in prevalence, particularly in Sub-Saharan Africa (133.50%), Eastern Europe (101.54%), Oceania (127.78%), and Australasia (114.70%; Table 1). In contrast, a significant decrease in the prevalence was observed in regions such as Southeast Asia, East Asia, High-income Asia Pacific, Tropical Latin America, Southern Sub-Saharan Africa, Western Europe and Central Europe, with East Asia decreasing by 33.77%, Western Europe by 24.36%, and Tropical Latin America by 20.18%. However, the AAPC exhibited a declining trend in most regions, with only five regions (Australasia, Oceania, Eastern Europe, Caribbean, and Central Asia) showing increase. Among them, Eastern Europe recorded the fastest increase, with the AAPC for ASPR of 2.05% (95% CI: 2.01 to 2.10; Table 1). East Asia experienced the fastest decline in ASPR (AAPC: -3.37, 95%CI: −3.45 to −3.33). In 2021, the region with the highest prevalence was South Asia, with 297,025 (95% UI: 250,483 to 347,641), followed by North Africa and the Middle East (63,633) and East Asia (73,595). In contrast, the regions with the lowest incidence rates were Australia (745) and Oceania (943).

In terms of YLDs, from 1990 to 2021, regions that exhibited a notable increase included Western Sub-Saharan Africa (102.69%), Australasia (100%), Oceania (90.91%), and High-income North America (60.34%). Compared with 1990, the YLDs in the East Asia, Tropical Latin America, and Central Europe regions showed a significant decrease in 2021, with a percentage of −50.57, −31.86%, and −30.18%, respectively. In 2021, the region with the highest YLDs was South Asia (10,744,95% UI: 6,745 to 15,932), followed by North Africa and the Middle East (1,701, 95% UI: 1089 to 2,514) and Western Sub-Saharan Africa (2,866, 95%UI: 1,835 to 4,250; Table 2). From the perspective of the changing trend of AAPC, most regions exhibited a decline trend, excepted for Eastern Europe with a AAPC of 1.52% (95% CI:1.47 to 1.57) and Caribbean with a AAPC of 0.3% (95% CI: 0.13 to 0.43). Of them, East Asia had the fastest declines in YLDs, with the AAPC of −4.34% (95% CI: −4.41 to −4.29).

### 3.3 Age and gender differences analysis

Compared with 1990, the prevalence of anemia associated with cirrhosis increased in both sexes in 2021, rising from 347,983 (95% UI: 299,528 to 399,671) to 415,550 (95% UI: 362,155 to 470,525) in females and from 296,290 (95% UI: 257,705 to 338,603) to 396,388 (95% UI: 347,237 to 447,612) in males (Table 1). Following age standardization, a significant downward trend was observed in prevalence for both sexes, with the ASPR declined from 13.48 to 10.32 per 100,000 in females and from 12.6 to 9.9 per 100,000 in males between 1990 and 2021. Correspondingly, the AAPCs were −0.82% (95% CI: −0.87 to −0.77) for females and −0.77% (95% CI: −0.83 to −0.71) for males. Regarding the disease burden, the absolute YLDs also showed a modest increase from 1990 to 2021, rising from 13,681 to 15,077 in females and from 7,365 to 7,661 in males. Nevertheless, the age-standardized YLD rates demonstrated a significant decline, consistent with the trend observed for prevalence. The rates declined from 0.52 to 0.38 per 100,000 for females and from 0.30 to 0.20 per 100,000 for males, with the AAPCs of −0.95% (95% CI: −1.01 to −0.88) for females and −1.34% (95% CI: −1.41 to −1.26) for males.

**Figure 1:**
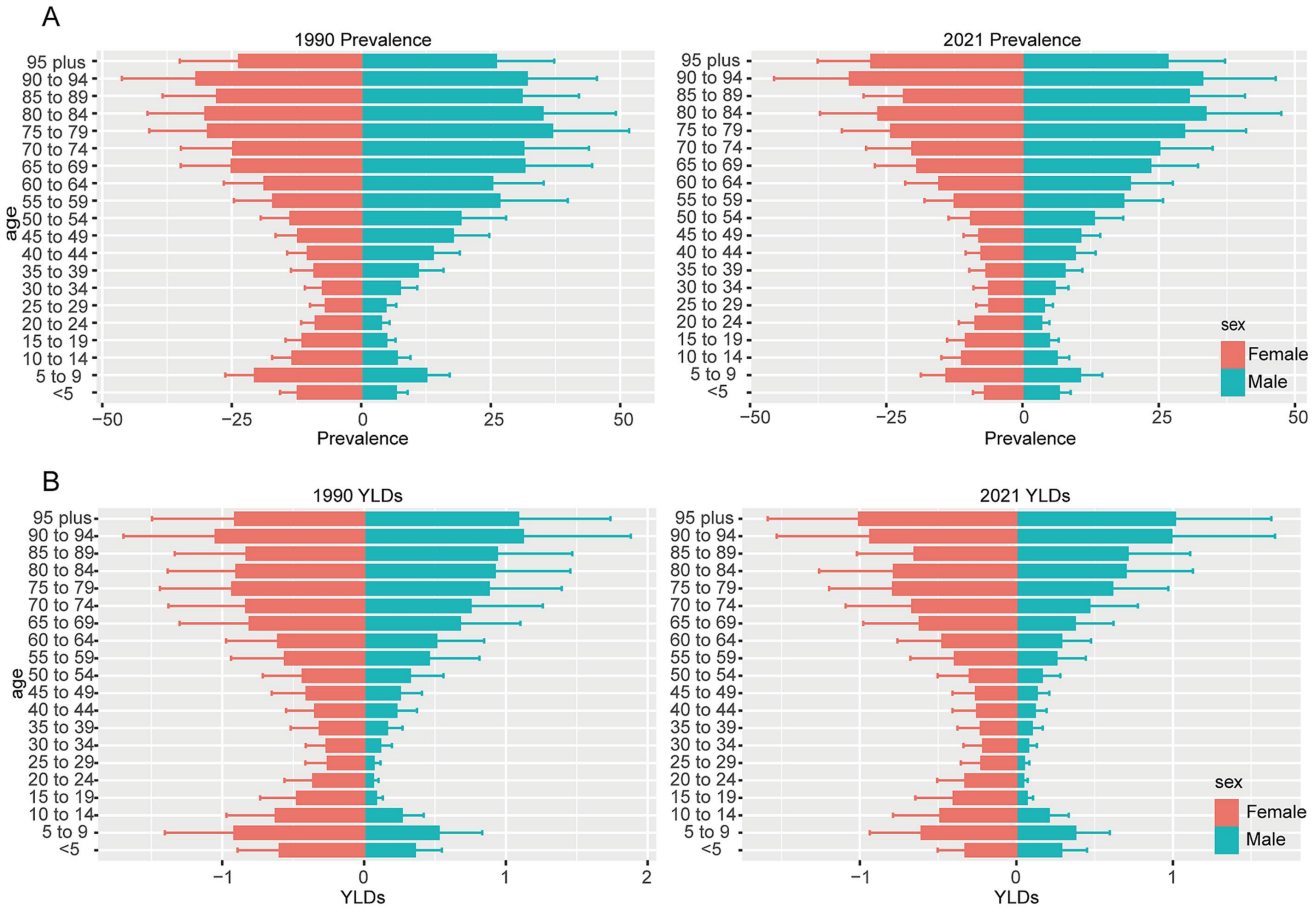
The prevalence **(A)** and YLDs **(B)** of anemia associated with liver cirrhosis in different genders and ages in 1990 and 2021.

In terms of age distribution, in 2021, both male and female exhibited the highest prevalence in the 90 to 94 age group (Figure 1A). However, in 1990, the prevalence rate was the highest among male in the 75 to 79 age group and among female in the 90 to 94 age group. For YLDs, man and women aged 90-plus years had the highest burden, differing from the 1990 data, which showed the highest burden in 90 to 94 age group (Figure 1B).

### 3.4 Decomposition analysis

**Figure 2:**
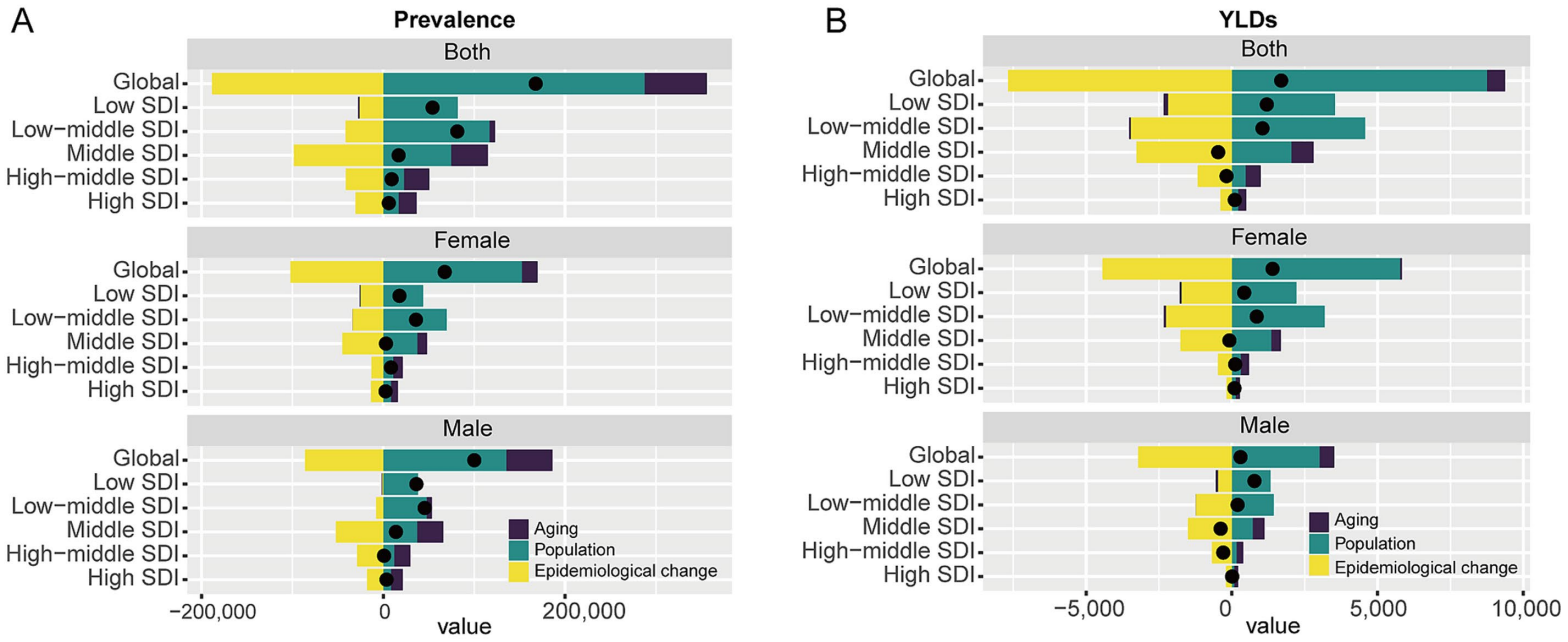
Decomposition analysis of prevalence and YLDs due to anemia attributed to liver cirrhosis worldwide, 1990–2021. **(A)** Changes in global and SDI quintile- specific prevalence for anemia associated with liver cirrhosis. **(B)** Changes in global and SDI quintile-specific YLDs rates for anemia associated with liver cirrhosis. SDI, sociodemographic index.

This study conducted a decomposition analysis to quantify the contributions of aging, population growth, and epidemiological changes to the trends in prevalence and YLDs of cirrhosis-related anemia from 1990 to 2021. The overall differences in prevalence rates are on the rise globally and in all SDI regions, with the most significant increase in the central SDI region (SupplementaryTable 1; Figure 2A). Globally, population aging and population growth had led to 40.88 and 171.48% increase in prevalence burden in the past 30 years, while epidemiological changes had a protective effect at −112.36%. The contribution of population growth to the prevalence burden varied across SDI regions, with the largest share observed in the middle-SDI (442.81%), followed by the high SDI region (276.64%). Population aging was also one of the primary drivers behind the rise in prevalence burden in SDI regions, except for low-SDI region (−3.10%). The contribution of aging in prevalence was more pronounced in higher-SDI regions, specifically high-SDI (330.24%). Epidemiological changes contributed to the reduction in global disease burden, especially in middle-SDI regions, at −583.81%. The overall difference showed an increased trend between males and females, with a more pronounced increase observed among males. For females, population aging negative growth occurred only in low SDI and low-middle SDI regions (−6.03% and −0.49%, respectively). Population growth accounted for the largest share of the increase in prevalence among females in middle SDI regions (1,268.91%), whereas among males, its contribution was greatest in high-middle SDI regions (1,514.71%). Consistently for both man and women, epidemiological changes were associated with a reduction in the prevalence burden.

For YLDs, from 1990 to 2021, the overall disease burden in the global, low SDI, low-middle SDI and high SDI regions increased, and only showed a decreasing trend in middle SDI and high-middle SDI regions (Supplementary Table 2; Figure 2B). Population aging contributes to the increase in disease sharing globally and in high SDI regions, accounting for 36.72 and 279.5%, respectively. In other regions, population aging has led to a reduction in the burden of YLDs, with the high-middle SDI has the greatest impact, at −280.94%. Overall, population growth has made a significant contribution to the increased burden of YLDs in most regions, while it seems to have a certain protective effect in middle SDI and high-middle SDI. The contribution of epidemiological changes to the change in the burden of YLDS in the middle SDI and high-middle SDI regions was 695.26 and 637.70%, respectively. However, for other regions, epidemiological changes are beneficial for reducing the burden of YLDS. Globally, population growth and epidemiological changes have a significant impact on the burden of YLDs in both men and women. Except for the middle SDI region, the burden of YLD for women has increased. In middle SDI region, population growth emerges as a negative contributing factor to the change in the YLD burden for females, while in other regions it does increase the burden of YLDs. Epidemiological changes have contributed to reducing the burden of YLD among women in most regions, except for Middle SDI. The disease burden of men in the middle SDI and high-middle SDI regions is reduced. In the high SDI region, population aging and population growth have increased the burden of male YLDs most significantly.

### 3.5 Temporal trends analysis

**Figure 3:**
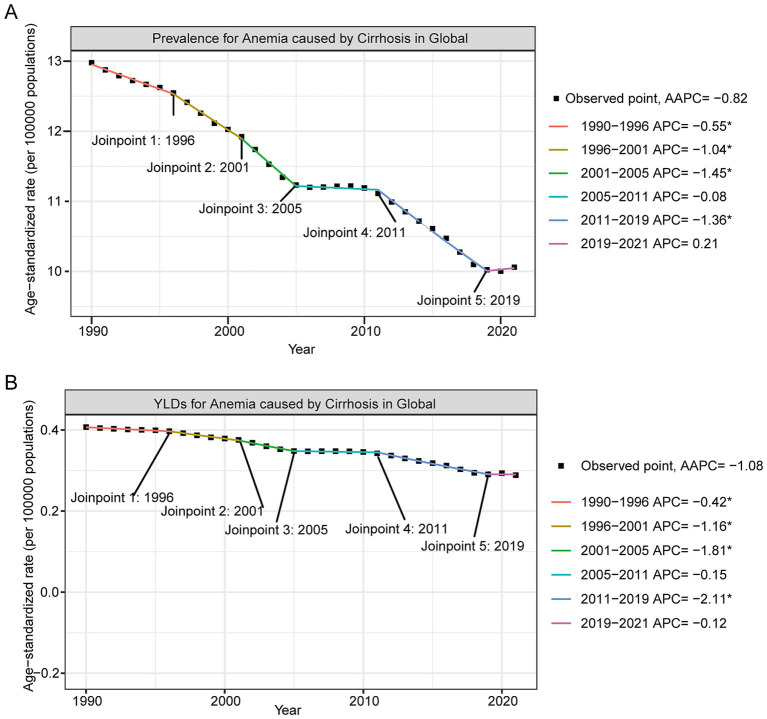
The joinpoint regression analysis of age-standardized rate of prevalence **(A)** and YLDs **(B)** from 1990 to 2021. APC, annual percentage change; AAPC, average annual percent change.

Joinpoint regression analyses was used to explore the change of ASPR and ASYR in global from 1990 to 2021. There was a decreasing trend from 1990 to 2001 (APC 1990–1996: -0.55, APC 1996–2001: −1.04) for ASPR (Supplementary Table 3; Figure 3A). Following a marked decline between 2001 and 2005 (APC: −1.45), the ASPR stabilized from 2005 to 2011 (APC: −0.08). Subsequently, a significant reduction occurred for ASPR from 2011 to 2019 (APC: −1.36), before a slight increase was noted from 2019 to 2021 (APC: 0.21). In summary, a significant decreasing trend in the global ASPR of anemia caused by cirrhosis was evident over the period 1990 to 2021 (Figure 3A). Similarly, from 1990 to 2021, the ASYR also showed a downward trend. Among them, the most significant decreases occurred from 2001 to 2005 and from 2011 to 2019, with APC values of −1.81 and −2.11, respectively (Figure 3B). Furthermore, we set the maximum number of joinpoints to 3, the location of joinpoints and the estimated APCs remained largely unchanged across these settings (Supplementary Figure S1). The overall trend was consistent, indicating that our conclusions are not sensitive to the choice of maximum joinpoints.

**Figure 4:**
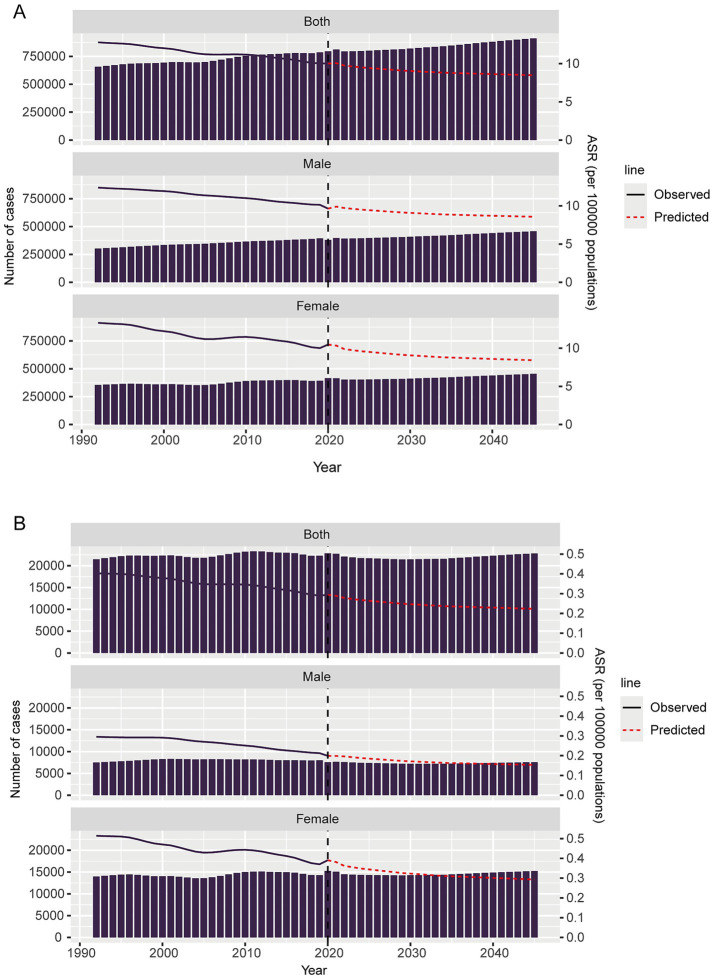
Predict the absolute and age-standardized prevalence **(A)** and YLDs **(B)** of anemia associated with anemia by Nordpred.

The prevalence and YLDs of anemia associated with liver cirrhosis from 2021 to 2045 were predicted through Nordpred analysis. Overall, during this period, the prevalence for both men and women showed a slight upward trend, but the age-standardized prevalence all showed a decreasing trend, and the decrease in women was greater than that in men (Figure 4A). For YLDs, it was predicted that by 2045, the YLDs of men would remain relatively stable, while that of women would increase slightly (Figure 4B). Consistent with the prediction results of Nordpred, the prediction results of BAPC also show similar trends (Supplementary Figure S2). To assess the goodness of fit of the model, we conducted a time-backward test verification. The verification results indicated that the observed values were in good agreement with the predicted values (Supplementary Table 4; Supplementary Figure S3). However, the age-standardized YLDs showed a downward trend for both man and woman.

## 4 Discussion

Based on GBD2021 data, our study systematically revealed for the first time at the global and SDI stratification levels the disease burden trend and driving factors of anemia associated with liver cirrhosis from 1990 to 2021. Research had found that although the age-standardized prevalence rate and the YLDs had both decreased significantly, the absolute number of prevalence and YLDs had continued to rise, and the differences among regions at different levels of development had become increasingly prominent. A more in-depth population attribution decomposition suggests that the contradictory phenomenon was mainly driven by population growth and aging, while epidemiological changes were the key to slowing down this trend, but their positive effects were extremely unevenly distributed globally.

Global and regional trend revealed the complexity of the transformation of the disease burden. The decline in global age-standardized rates (ASPR AAPC: −0.82%; ASYR AAPC: −1.08%) could be attributed to advances made over the past three decades in viral hepatitis prevention and control (17, 18), alcohol-related policies (19, 20), improvements in healthcare coverage, and the management of anemia (21, 22). Even as medical advances lowered individual disease risk, the absolute burden grew substantially, with prevalence and YLDs increasing by 26.0 and 8.0%, respectively; this trend underscores that global population growth has partially negated these risk-reduction benefits. This is a common predicament in the prevention and control of non-communicable diseases (23, 24). Of particular note was the marked regional heterogeneity: areas with steep declines, such as East Asia, Southeast Asia, and Western Europe (e.g., East Asia’s ASPR AAPC reached −3.37%), stood in sharp contrast to regions where the burden increased rather than declined, including Eastern Europe, Central Asia, and the Caribbean (e.g., Eastern Europe’s ASPR AAPC was +2.05%). The latter finding may imply that growing prevalence of alcohol-related liver disease, metabolic syndrome, or certain infectious contributors could potentially offset global improvements in disease burden, which highlights a potential need for targeted etiological research and tailored public health interventions.

The level of socioeconomic development emerged as a key determinant of the distribution and temporal trends in disease burden (25–27). In high SDI regions, although the age-standardized prevalence rate showed a declining trend, the absolute number of prevalent cases continued to rise. The main reason for this phenomenon is that the survival period of patients with liver cirrhosis has been improved. The growing number of long-term surviving patients leads to higher absolute case numbers, even as the overall population prevalence rate declines. SDI analysis further revealed that there was a clear gradient in the age-standardized burden, with the highest levels in regions with medium to low SDI and below. Importantly, the rate at which the disease burden decreases were positively correlated with the level of SDI. For instance, the annual decline rate of ASPR in middle and high SDI regions (AAPC approximately −1.3% to −1.4%) was significantly faster than that in low SDI regions (−0.76%). While this disparity might be partially attributable to differences in healthcare infrastructure and accessibility, these factors were not directly assessed in our study; thus, this interpretation remains hypothetical rather than conclusive. Meanwhile, the moderate increase in the prevalence in high SDI regions (such as an 8.4% increase in prevalence of high SDI cases) contrasts sharply with the sharp increase in low SDI regions (a 72.00% increase in the number of low SDI cases), highlighting the global health inequality.

Gender analysis shows that women bear a heavier burden in terms of age-standardized rates, which may be related to their physiological susceptibility to anemia (28–30), such as menstrual blood loss and pregnancy. However, the absolute increase in prevalence of man patients was greater (33.80% for men and 19.40% for women), which might be related to the higher incidence of liver cirrhosis (especially alcoholic) among men (31–33) and the slightly faster growth of the global man population (34). This study innovatively quantified the independent contributions of the three major driving factors through the method of population attribution decomposition (35). Data showed that population growth was the overall and primary driving force for the increase in the prevalence (contributing 171.48% globally to prevalence cases). The role of population aging was particularly prominent in regions with high SDI (contribution 330.24%), demonstrating the pressure on the chronic disease prevention and control system in a deeply aging society. Epidemiological changes were the only negative factor that curbed the growth (global contribution −112.36%). However, its offsetting capacity was seriously insufficient in low SDI regions (contributing only −48.09%), while it was powerful in high SDI regions (contributing −506.88%). This suggests that the level of development not only determined the distribution of diseases, but also affected whether society could fully utilize medical progress to address the health challenges brought about by the transformation of population structure.

It was crucial to acknowledge that there were several limitations in our study. Firstly, the diagnosis of “anemia associated with cirrhosis” involves complexity in real-world settings, and the strength of association may have been oversimplified in the modeling. Secondly, the decomposition analysis did not incorporate specific policy and environmental factors such as healthcare accessibility and economic fluctuations. Finally, the lack of subgroup analysis by etiology of cirrhosis (viral, alcoholic, non-alcoholic fatty liver disease) limited the precision with which intervention strategies could be targeted.

Despite certain limitations, our findings may provide clear implications for public health practice. Firstly, high-income countries globally should integrate anemia management into comprehensive liver disease care systems to address the increasing case burden driven by population aging. Secondly, low-income and lower-middle-income countries should focus on primary etiological prevention and control by strengthening hepatitis immunization, improving treatment accessibility, curbing alcohol-related harm, and enhancing primary-level anemia diagnosis and treatment capacity to accelerate the reduction of disease burden. Thirdly, regions with rising burden, such as Eastern Europe and Central Asia, should conduct targeted investigations to identify key risk factors (e.g., heavy alcohol consumption, obesity prevalence, healthcare system gaps) and implement precise interventions. Finally, in clinical practice, routine anemia screening and comprehensive management for cirrhosis patients—particularly males—should be strengthened to improve prognosis and quality of life.

## 5 Conclusion

Globally, progress has been made in reducing the age-standardized risk of anemia associated with cirrhosis. However, this progress is being weakened by population growth and structural transitions, and may be associated with substantial regional inequality. On one hand, in the future, efforts should continue to be made to promote primary prevention and early treatment based on etiology, and consolidate the positive trend of epidemiological change. On the other hand, health resources should be allocated in a forward-looking manner based on the population structure and development stage of each region to address the systemic pressure brought about by the rising absolute disease burden, thereby promoting the global management of liver diseases and anemia toward a fairer and efficient direction.

## Data Availability

The original contributions presented in the study are included in the article/Supplementary material, further inquiries can be directed to the corresponding author.

## Footnotes

1
http://ghdx.healthdata.org/gbd-results-tool
