# Supplementary material for: Trends in the global burden of anemia associated with liver cirrhosis: an assessment from 1990 to 2021 and projections to 2045
Source: Front Public Health. 2026 Jul 15;14:1845819. doi: 10.3389/fpubh.2026.1845819 (PMC13414119; doi:10.3389/fpubh.2026.1845819)
Supplement: Supplementary file 1 [file Data_Sheet_1.docx]

**Supplementary Table 1** Changes of prevalence in anemia associated with cirrhosis from 1990 to 2021, decomposed by population-level determinants: population aging, population growth and epidemiological changes

| Location | Both | | | |  | Female | | | |  | Male | | | |
| --- | --- | --- | --- | --- | --- | --- | --- | --- | --- | --- | --- | --- | --- | --- |
|  | Overall difference | Aging | Population | Epidemiological change |  | Overall difference | Aging | Population | Epidemiological change |  | Overall difference | Aging | Population | Epidemiological change |
| Global | 167664.52 | 68535.9 (40.88%) | 287512.37 (171.48%) | -188383.75 (-112.36%) |  | 67566.42 | 17321.63 (25.64%) | 152414.97 (225.58%) | -102170.18 (-151.21%) |  | 100098.11 | 50755.49 (50.71%) | 135256.84 (135.12%) | -85914.22 (-85.83%) |
|  |  |  |  |  |  |  |  |  |  |  |  |  |  |  |
| Low SDI | 54162.38 | -1680.69 (-3.1%) | 81887.77 (151.19%) | -26044.7 (-48.09%) |  | 17783.03 | -1072.99 (-6.03%) | 43793.63 (246.27%) | -24937.6 (-140.23%) |  | 36379.35 | -753.32 (-2.07%) | 38162.6 (104.9%) | -1029.94 (-2.83%) |
| Low-middle SDI | 81375.8 | 6066.69 (7.46%) | 116866.44 (143.61%) | -41557.34 (-51.07%) |  | 35922.17 | -176.48 (-0.49%) | 69726.16 (194.1%) | -33627.51 (-93.61%) |  | 45453.63 | 5631.04 (12.39%) | 47719.16 (104.98%) | -7896.57 (-17.37%) |
|  |  |  |  |  |  |  |  |  |  |  |  |  |  |  |
| Middle SDI | 16831.29 | 40564.03 (241%) | 74530.4 (442.81%) | -98263.14 (-583.81%) |  | 2938.53 | 10704.4 (364.28%) | 37287.26 (1268.91%) | -45053.13 (-1533.19%) |  | 13892.76 | 28883.89 (207.91%) | 37169.59 (267.55%) | -52160.72 (-375.45%) |
| High-middle SDI | 9186.35 | 27853.54 (303.21%) | 22700.04 (247.11%) | -41367.22 (-450.31%) |  | 8401.28 | 10483.55 (124.79%) | 10846.24 (129.1%) | -12928.51 (-153.89%) |  | 785.07 | 17761.37 (2262.39%) | 11891.51 (1514.71%) | -28867.81 (-3677.1%) |
|  |  |  |  |  |  |  |  |  |  |  |  |  |  |  |
| High SDI | 6034.46 | 19928.03 (330.24%) | 16694.02 (276.64%) | -30587.59 (-506.88%) |  | 2493.24 | 7751.95 (310.92%) | 8318.59 (333.65%) | -13577.29 (-544.56%) |  | 3541.22 | 12940.93 (365.44%) | 8356.92 (235.99%) | -17756.64 (-501.43%) |

**Supplementary Table 2** Changes of YLDs in anemia associated with cirrhosis from 1990 to 2021, decomposed by population-level determinants: population aging, population growth and epidemiological changes

| Location | Both | | | |  | Female | | | |  | Male | | | |
| --- | --- | --- | --- | --- | --- | --- | --- | --- | --- | --- | --- | --- | --- | --- |
|  | Overall difference | Aging | Population | Epidemiological change |  | Overall difference | Aging | Population | Epidemiological change |  | Overall difference | Aging | Population | Epidemiological change |
| Global | 1691.64 | 621.14 (36.72%) | 8751.59 (517.35%) | -7681.1 (-454.06%) |  | 1395.36 | 61.16 (4.38%) | 5770.78 (413.57%) | -4436.58 (-317.95%) |  | 296.28 | 506 (170.78%) | 3007.42 (1015.06%) | -3217.14 (-1085.84%) |
|  |  |  |  |  |  |  |  |  |  |  |  |  |  |  |
| Low SDI | 1197.55 | -156.25 (-13.05%) | 3536.27 (295.29%) | -2182.47 (-182.24%) |  | 423.59 | -73.93 (-17.45%) | 2216.94 (523.37%) | -1719.42 (-405.92%) |  | 773.96 | -85.93 (-11.1%) | 1327.25 (171.49%) | -467.36 (-60.38%) |
| Low-middle SDI | 1049.31 | -59.69 (-5.69%) | 4572.66 (435.78%) | -3463.66 (-330.09%) |  | 852.24 | -78.34 (-9.19%) | 3184.05 (373.61%) | -2253.47 (-264.42%) |  | 197.07 | -2.54 (-1.29%) | 1433.94 (727.62%) | -1234.32 (-626.33%) |
|  |  |  |  |  |  |  |  |  |  |  |  |  |  |  |
| Middle SDI | -470.95 | 761.11 (-161.61%) | 2042.27 (-433.65%) | -3274.32 (695.26%) |  | -85.84 | 327.76 (-381.83%) | 1349.11 (-1571.66%) | -1762.71 (2053.48%) |  | -385.11 | 409.38 (-106.3%) | 712.09 (-184.91%) | -1506.58 (391.21%) |
| High-middle SDI | -183.54 | 515.63 (-280.94%) | 471.25 (-256.76%) | -1170.42 (637.7%) |  | 114.4 | 287.25 (251.1%) | 304.25 (265.96%) | -477.11 (-417.06%) |  | -297.93 | 221 (-74.18%) | 163.3 (-54.81%) | -682.23 (228.99%) |
|  |  |  |  |  |  |  |  |  |  |  |  |  |  |  |
| High SDI | 99.11 | 277 (279.5%) | 214.72 (216.66%) | -392.62 (-396.15%) |  | 90.57 | 142 (156.78%) | 133.19 (147.05%) | -184.61 (-203.83%) |  | 8.54 | 135.08 (1582.26%) | 78.11 (914.97%) | -204.65 (-2397.24%) |

**Supplementary Table 3** Joinpoint regression analysis: trends in age-standardized prevalence, YLDs rates (per 100,000 persons) in global, 1990–2021

| **Period** | **Prevalence** |  |  | **YLDs** |  |
| --- | --- | --- | --- | --- | --- |
|  | APC (95% CI) | P value |  | APC (95% CI) | P value |
| 1990-1996 | -0.55  (-0.67 to -0.37) | 0.01 |  | -0.42 (-0.61 to -0.04) | 0.04 |
| 1996-2001 | -1.04  (-1.3 to -0.77) | 0 |  | -1.16 (-1.61 to -0.63) | 0.01 |
| 2001-2005 | -1.45  (-1.67 to -0.1) | 0.02 |  | -1.81 (-2.2 to -0.08) | 0.04 |
| 2005-2011 | -0.08  (-1.17 to 0.06) | 0.12 |  | -0.15 (-1.95 to 0.07) | 0.11 |
| 2011-2019 | -1.36  (-1.46 to -1.29) | 0 |  | -2.11 (-2.3 to -1.99) | 0 |
| 2019-2021 | 0.21  (-0.26 to 0.49) | 0.22 |  | -0.12 (-0.88 to 0.38) | 0.62 |

APC annual percent change

**Supplementary Table 4** The Nordpred model fitting evaluation indicators

|  | MAPE | R^2^ |
| --- | --- | --- |
| Prevalence | 7.31% | 0.975 |
| YLDs | 9.24% | 0.966 |

MAPE Mean Absolute Percentage Error

**Supplementary Figure S1**

**
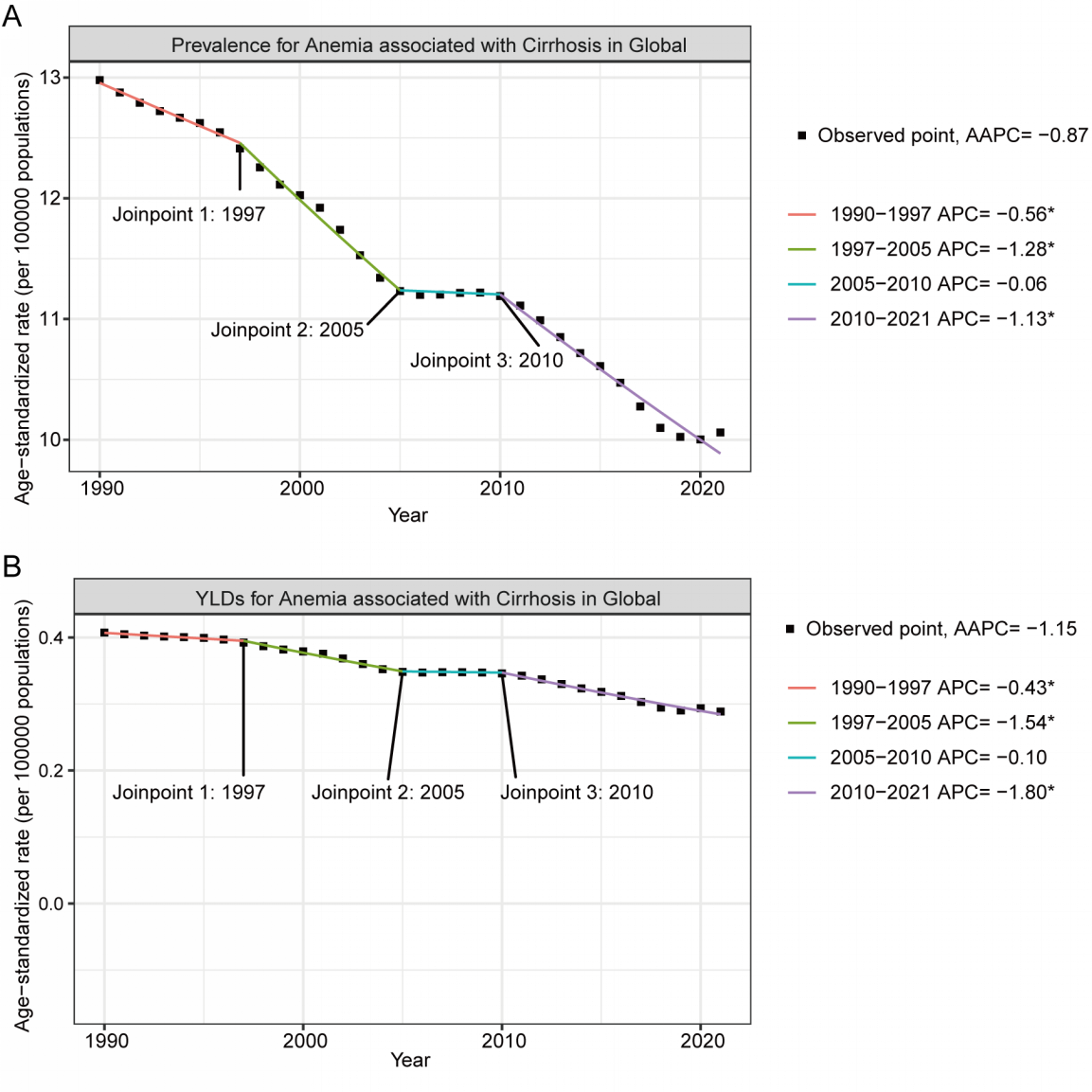
**

**Supplementary Figure S1**  The joinpoint regression analysis of age-standardized rate of prevalence (A) and YLDs (B) from 1990 to 2021. APC, annual percentage change; AAPC, average annual percent change.

**Supplementary Figure S2**

**
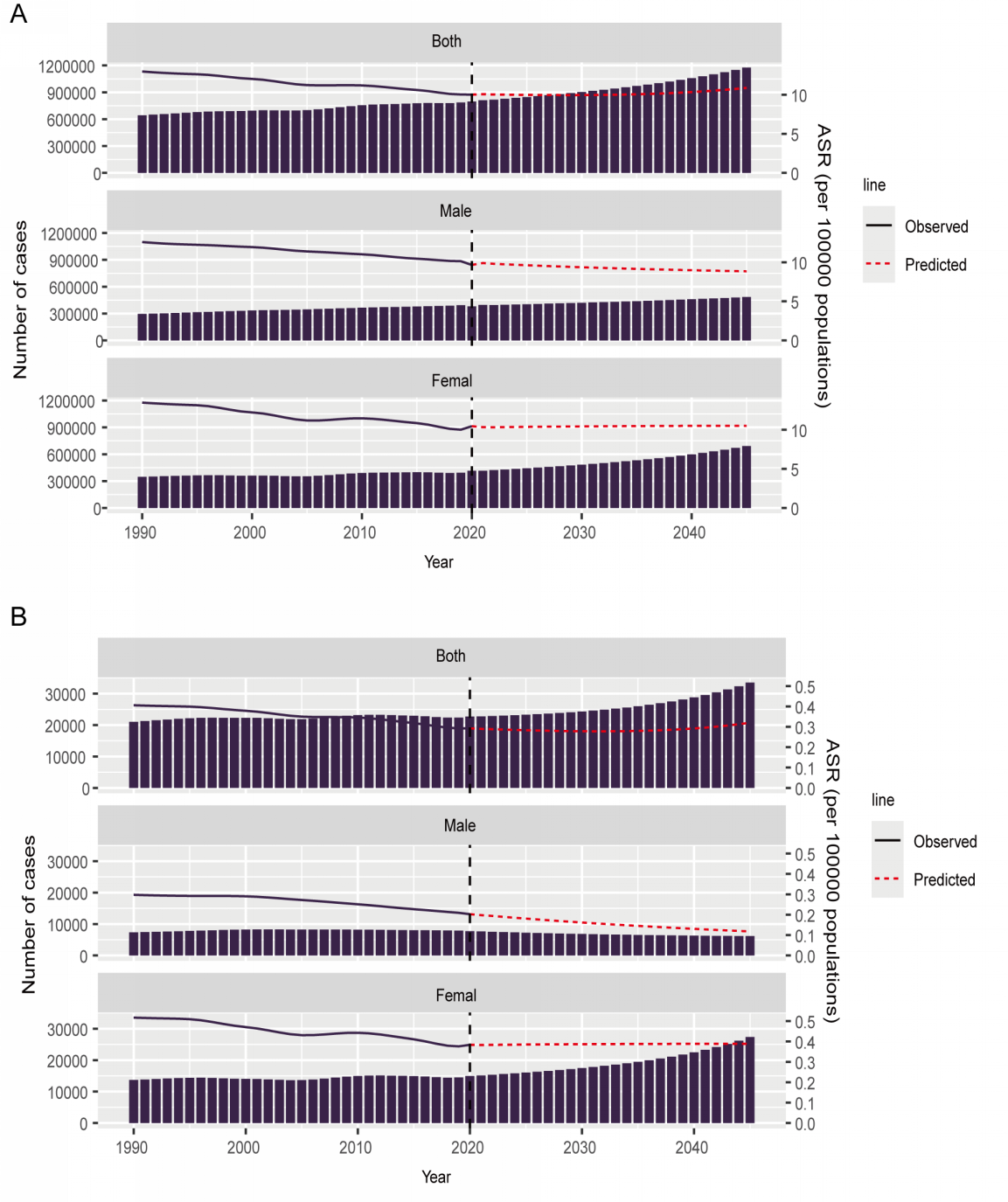
**

**Supplementary Figure S2** Predict the absolute and age-standardized prevalence (A) and YLDs (B) of anemia associated with anemia by BAPC.

**Supplementary Figure S3**

**
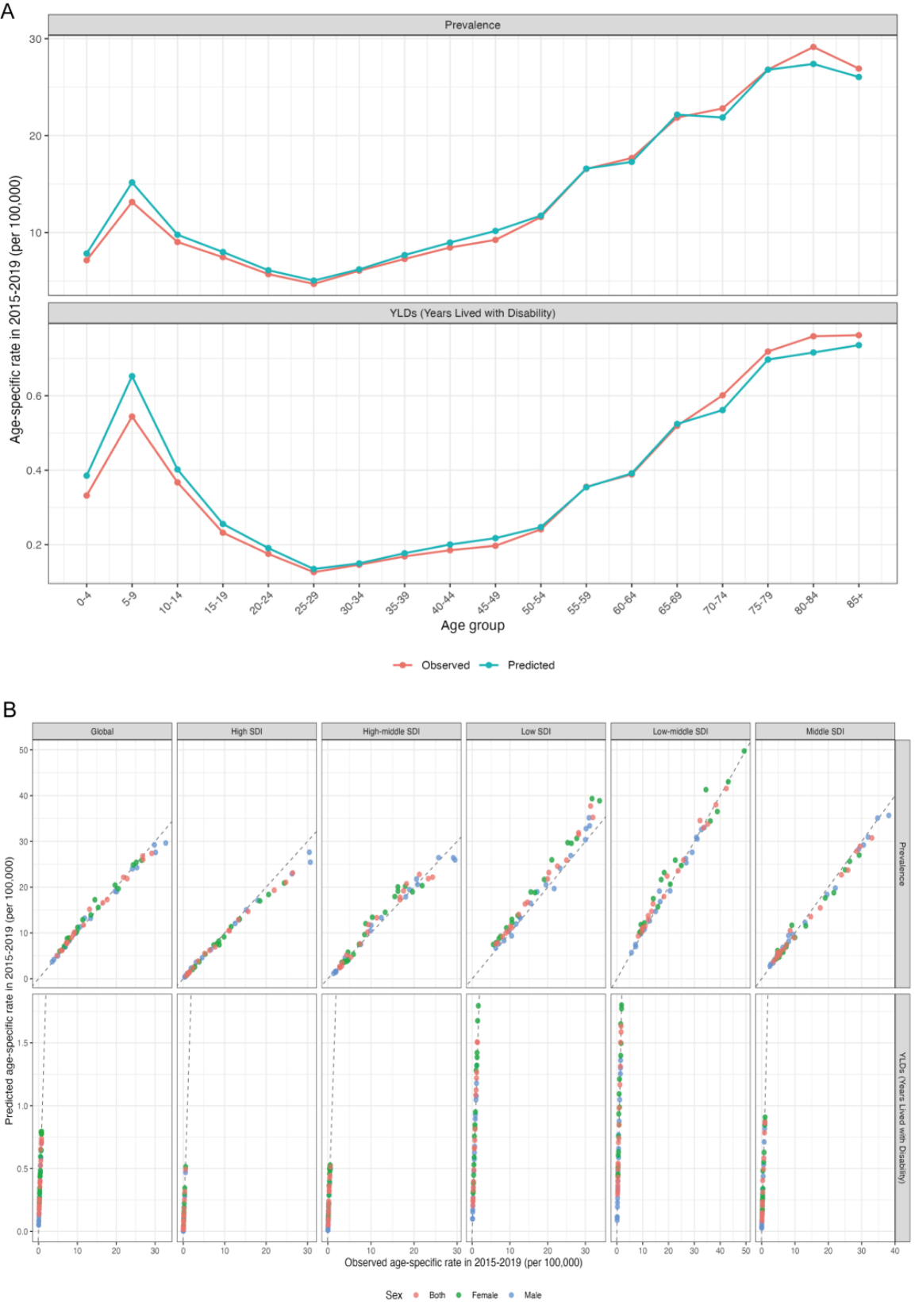
**

**Supplementary Figure S3** Observed versus Nordpred-predicted age-specific prevalence and YLDs across age groups (A). Agreement between Nordpred predicted and GBD-observed age-specific prevalence and YLD rates across global and five SDI categories by sex (B).
